# The Protective Role of Cranberries and Blueberries in Oral Cancer

**DOI:** 10.3390/plants12122330

**Published:** 2023-06-15

**Authors:** César Esquivel-Chirino, Mario Augusto Bolaños-Carrillo, Daniela Carmona-Ruiz, Ambar Lopéz-Macay, Fernando Hernández-Sánchez, Delina Montés-Sánchez, Montserrat Escuadra-Landeros, Luis Alberto Gaitán-Cepeda, Silvia Maldonado-Frías, Beatriz Raquel Yáñez-Ocampo, José Luis Ventura-Gallegos, Hugo Laparra-Escareño, Claudia Patricia Mejía-Velázquez, Alejandro Zentella-Dehesa

**Affiliations:** 1 Área de Básicas Médicas, División de Estudios Profesionales, Facultad de Odontología, Universidad Nacional Autónoma de México, Ciudad de México 04510, Mexico; 2 Área de Ciencias Naturales, Departamento de Bachillerato, Universidad del Valle de México, Campus Guadalajara Sur, Guadalajara 045601, Mexico; mario_bolanos@my.uvm.edu.mx; 3 Área de Ortodoncia, División de Estudios Profesionales, Facultad de Odontología, Universidad Nacional Autónoma de México, Ciudad de México 04510, Mexico; 4 Laboratorio de Liquído Sinovial, Instituto Nacional de Rehabilitación LGII, Ciudad de México 14389, Mexico; 5 Departamento de Virología y Micología, Instituto Nacional de Enfermedades Respiratorias “Ismael Cosío Villegas”, Ciudad de México 04502, Mexico; 6 Investigación Biomédica Básica, Licenciatura en Estomatología, Benemérita Universidad Autónoma de Puebla, Puebla 75770, Mexico; 7 Facultad de Odontología, Universidad Intercontinental, Ciudad de México 14420, Mexico; 8 Departamento de Medicina y Patología Oral Clínica, División de Estudios de Posgrado e Investigación, Facultad de Odontología, Universidad Nacional Autónoma de México, Ciudad de México 04510, Mexico; 9 Laboratorio de Bioingeniería de Tejidos, División de Estudios de Posgrado e Investigación, Facultad de Odontología, Universidad Nacional Autónoma de México, Ciudad de México 04360, Mexico; sylvymaf@comunidad.unam.mx; 10 Especialidad en Periodoncia e Implantología, División de Estudios de Posgrado e Investigación, Facultad de Odontología, Universidad Nacional Autónoma de México, Ciudad de México 04510, Mexico; 11 Departamento de Medicina Genómica y Toxicología Ambiental, Instituto de Investigaciones Biomédicas, UNAM, Ciudad de México 04510, Mexico; 12 Departamento de Cirugía, Sección de Cirugía Vascular y Terapia, Instituto de Ciencias Médicas y Nutrición Salvador Zubirán, Ciudad de México 14080, Mexico; 13 Departamento de Patología, Medicina Bucal y Maxilofacial, Facultad de Odontología, Universidad Nacional Autónoma de México, Ciudad de México 04510, Mexico; 14 Unidad de Bioquímica, Instituto de Ciencias Médicas y Nutrición Salvador Zubirán, Ciudad de México 14080, Mexico

**Keywords:** oral cancer, oral squamous carcinoma cell (OSCC), phytochemicals, berries, flavonoids, flavanols, cranberries, blueberries

## Abstract

Background: Oral cancer has a high prevalence worldwide, and this disease is caused by genetic, immunological, and environmental factors. The main risk factors associated with oral cancer are smoking and alcohol. Results: There are various strategies to reduce risk factors, including prevention programs as well as the consumption of an adequate diet that includes phytochemical compounds derived from cranberries (*Vaccinium macrocarpon* A.) and blueberries (*Vaccinium corymbosum* L.); these compounds exhibit antitumor properties. Results: The main outcome of this review is as follows: the properties of phytochemicals derived from cranberries were evaluated for protection against risk factors associated with oral cancer. Conclusions: The secondary metabolites of cranberries promote biological effects that provide protection against smoking and alcoholism. An alternative for the prevention of oral cancer can be the consumption of these cranberries and blueberries.

## 1. Background

Cancer is a disease characterized by the proliferation of tumor cells, followed by the invasion of distant organs (metastasis), and can cause serious health complications, often leading to the death of the patient [1,2,3,4,5]. According to statistics from 2020, approximately 10 million people in the world died from cancer, and one in every six deaths was attributed to this disease. A significant percentage of cases correspond to oral cancer, which affects anatomical regions, such as the base of the tongue, lip, gingiva, tonsil, floor of the mouth, and uvula [6,7,8]. Oral cancer accounts for 85% of neoplasms in the oral cavity [9].

Approximately 90% of oral cancers correspond to oral squamous cell carcinoma, while the remaining 10% are classified as melanomas, sarcomas, minor salivary gland carcinomas, or metastatic carcinomas [10]. Oral cancer survival is reported to average five years [11,12]. The cause of oral cancer involves several factors, including genetic, environmental, immunological, and viral infections, and contact mainly with tobacco and alcohol consumption [6]. An inadequate daily diet, especially a low consumption of fruits and vegetables, is another important factor associated with cancer progression [13,14]. A balanced diet should include compounds derived from plant and fruit extracts, such as phytochemicals, phenolics, and flavonoids, to obtain their antioxidant and protective properties against various risk factors, as well as their antitumor effects [15,16,17]. Furthermore, the use of phytochemicals has been identified as a possible protective factor against oral cancer, particularly in fruits of the *Ericaceae* family, such as cranberries and blueberries [18].

Berries contain phytochemical compounds with biological activity, such as flavanols, ellagitannins, gallotannins, proanthocyanidins, and anthocyanins [15,16,17]. The antioxidant and antitumor properties of berry phytochemicals have been shown in vitro and in vivo in oral cancer models [17,18,19,20,21]. The aim of this work is to investigate the antioxidant activities of berry and cranberry phytochemicals in protecting the oral mucosa against oral cancer risk factors.

## 2. Materials and Methods

A comprehensive literature search was conducted from March 2000 to March 2023. Keyword searches were conducted in databases, such as PubMed, EBSCO, Wiley, and SpringerLink, using terms, such as “oral cancer,”, “berries”, “oral cancer and berries”, “phytochemicals and oral cancer”, and “cranberries and oral cancer.” Articles were selected if they contained information about phytochemicals and cancer. Studies that mentioned effects on other types of cancer were also included. The exclusion criteria were articles that were not written in English and articles from popular science journals and dissertations.

## 3. Introduction

### 3.1. Head and Neck Cancer

**Figure 1:**
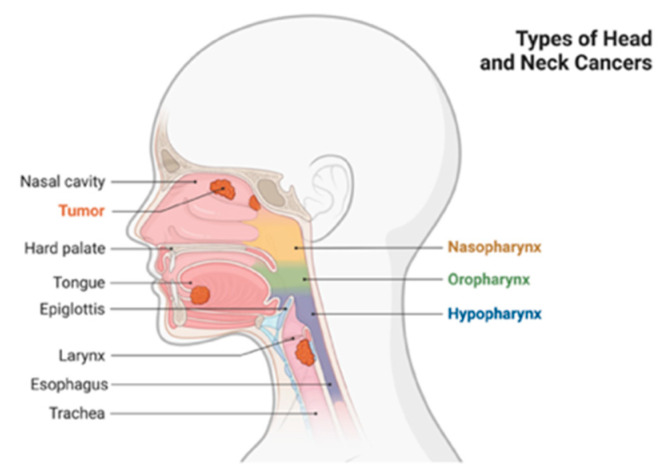
Head and neck cancer distribution. Created with BioRender.com (accessed on 10 March 2022).

This neoplasm involves the oral cavity, larynx, oropharynx, and paranasal sinuses (Figure 1) [21,22].

### 3.2. Oral Cancer

**Figure 2:**
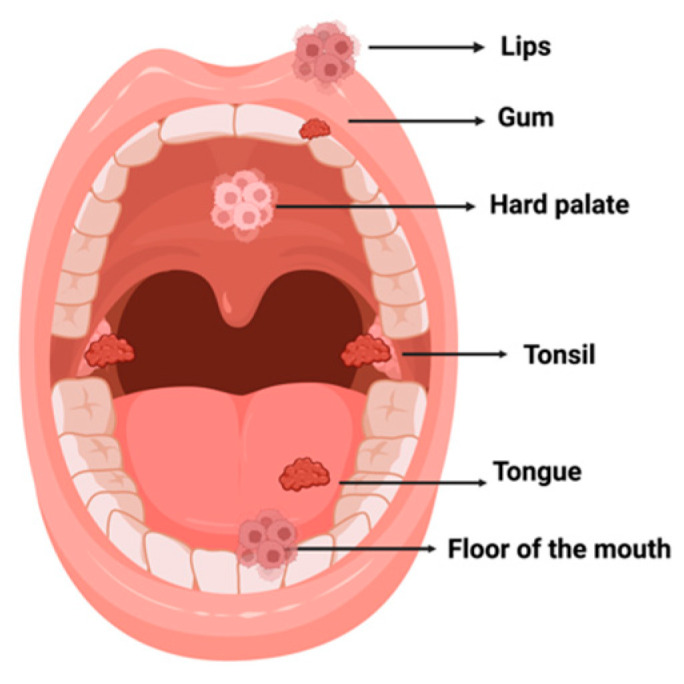
Distribution of oral cancer. Created with BioRender.com (accessed on 10 March 2022).

Oral cancer is defined as a malignant neoplasm that can manifest in any part of the oral cavity [21]. The most frequent anatomical sites for oral cancer are the tongue (including the base and anterior part), gums, tonsils, oropharynx, lips, floor of the mouth, soft and hard palate, oral mucosa, and salivary glands (Figure 2) [22].

### 3.3. Epidemiology

Oral cancer represents the sixth-highest prevalence of malignant neoplasms in the developed world; however, it is the eighth most-prevalent malignant neoplasm in less-developed countries [23]. During 2020, 377,713 new cases of oral cancer were diagnosed worldwide, and of those cases, 177,757 resulted in death [24,25]. A retrospective study conducted between 1990 and 2019 reported that Asia had the highest number of oral cancer cases, followed by North America, South America, and Europe [26]. Oral cancer is 3.6 times more frequent among men than women, with higher mortality rates in underdeveloped countries [27].

### 3.4. Etiology and Risk Factors

The cause of oral cancer involves multiple factors; however, the main risk factors are tobacco and alcohol consumption [7,28]. Several risk factors have been identified for oral cancer, including viral infections, especially human papillomavirus (HPV), immunosuppression, genetic predisposition, poor oral hygiene, and an inadequate diet [26,27,29]. Another important factor associated with oral cancer is chronic inflammatory processes, such as periodontal disease [30,31].

#### 3.4.1. Tobacco

The use of tobacco products is the principal cause of more than 8 million cancer deaths worldwide [32]. Tobacco products have different carcinogenic properties [33]. People who smoke have a predisposition to oral cancer that is between seven and ten times higher than that of nonsmokers, although the level of exposure depends on the frequency and duration of smoking [34,35,36,37]. The most common carcinogens in tobacco are benzopyrenes, 4-(methylnitrosamine)-1-(3-pyridyl)-1-butanone, and N′-nitrosonornicotine (NNN) [38,39]. The metabolites of these compounds present in tobacco induce mutations that affect DNA replication and the genes involved in the control of cell growth, favoring damage to the oral mucosa and malignant transformation [40,41]. In Asia, a form of smokeless tobacco called Guthka is consumed, which is a mixture of betel nut (*Areca catechu*), tobacco, and spices. This preparation is popular among the young population due to its stimulating, relaxing effects [42]. However, betel nut contains tannins and alkaloids, such as arecoline, which have negative effects on health and have been associated with oral cancer [30,42]. Tobacco products have carcinogenic and genotoxic effects on epithelial cells and are related to pancreatic cancer, cardiovascular disease, periodontal conditions, and asthma [31]. In addition, tobacco consumption can contribute to the development of esophageal carcinoma [43]. Tobacco products generate reactive oxygen species (ROS), change pH, and cause mucosal irritation, activating T cells as well as macrophages, which promote prostaglandin production and hyperplasia [40]. These types of irritants and subsequent inflammation are important promoter events in the progression to malignant transformation.

#### 3.4.2. Alcohol

Alcohol consumption is a recognized factor in the development of oral and oropharyngeal cancer [12,40]. The main metabolite of alcohol is acetaldehyde, which is involved in DNA synthesis and repair and induces, among many other effects, the exchange of sister chromatids and mutations [40,41]. Alcohol acts as a local irritant chemical when in contact with the oral mucosa. Therefore, by dissolving and damaging the lipids of the epithelium, alcohol consumption increases the permeability of the oral mucosa [44]. Frequent alcohol consumption is associated with impaired innate and acquired immunity, which increases the susceptibility of the oral cavity to infections and neoplasms [45]. Alcohol is metabolized by enzymes, such as dehydrogenase, cytochrome P-4502 E1, and catalases, generating acetaldehyde. This metabolite has carcinogenic and genotoxic properties [46]. In addition, the consumption of alcohol activates inflammatory processes since inflammatory white cells are recruited and various interleukins are produced, which causes the formation of ROS [47]. It has been reported that the consumption of 10 g or more of alcohol per day has been related to a 15% increased risk of having oral cancer and a 10% increased risk of having pharyngeal cancer [48]. Oral squamous cell carcinoma is related to polymorphisms of the ALDH2 gene [49]. Alcohol and tobacco use increase the risk of oral cancer up to five-fold [50,51,52].

#### 3.4.3. Viral Infections

The major viral infections associated with oral cancer include human herpesvirus and human papillomavirus (HPV) [53,54]. These viruses cause genetic instability through mutations, aberrations, and DNA damage [55].

##### Human Papillomavirus

HPV is the virus most frequently associated with cases of oral cancer [56]. HPV genotypes 16 and 18 are the most frequently associated with oral cancer. HPV encodes two oncoproteins, E6 and E7, which bind to p53 and Rb proteins, causing the loss of regulation of DNA replication, repair, and apoptosis [57].

##### Epstein–Barr Virus

This virus presents double-stranded DNA with oncogenic potential and has been considered the causal agent of several neoplasms, including squamous oral cell carcinoma (OSCC) [58]. In 1997, this virus was recognized as the cause of nasopharyngeal carcinoma and has also been associated with Hodgkin lymphoma, NK cell lymphoma, and gastric carcinoma [59]. People infected with Epstein–Barr virus have a 2.5-fold increased probability of acquiring OSCC. However, the direct association between OSCC and Epstein–Barr virus is not completely clear [60].

#### 3.4.4. Oral Health

Poor oral hygiene can affect the oral microbiota by allowing bacteria to evade the host immune response and increase their growth, which can cause a shift from symbiosis to dysbiosis [61]. In addition, an increased amount of endogenous nitrosamine, a major carcinogen, is produced, which may pose a risk for oral cancer initiation [50]. A report found that, in patients with inadequate mouth hygiene, there was a seven-fold increased risk of developing oral cancer [51]. In addition, tooth fractures, tooth decay, and poorly fitting dentures can cause chronic irritation of the oral mucosa, which, in combination with other factors, such as smoking or drinking alcohol, may promote the occurrence of oral cancer [62]. Another relevant factor is the use of mouthwashes containing alcohol as a solvent or preservative in combination with tobacco or alcohol consumption [63,64]. However, there are no conclusive data to suggest an increased risk of oral cancer from the use of these mouthwashes alone.

#### 3.4.5. Diet and Nutrition

Several studies have found that low fruit and vegetable consumption may contribute to the increased risk of developing oral cancer [65]. A high body mass index (BMI) has been implicated in an elevated risk of developing oral cancer [66]. It is important to analyze the relationship between food consumption and the risk of oral cancer. To date, it is considered that an adequate diet should include the consumption of phytochemicals, phenolics, and flavonoids that have antioxidant and antitumor properties [16,17,18]. In addition, the consumption of phytochemicals has been reported to have protective effects against risk factors associated with oral cancer [18]. Therefore, it is recommended to consume foods that contain a significant concentration of phytochemicals, such as berries, the consumption of which has been related to a reduction in oxidative stress and inflammation [16].

### 3.5. Premalignant Lesions

One area of opportunity remains the early detection of oral cancer. Therefore, the development of programs and strategies for the early detection of premalignant lesions may improve patient survival and prognosis [67]. Therefore, a detailed patient history and a thorough clinical examination are essential [16]. Oral cancer patients have no symptoms in the early stages, leading to diagnosis in advanced stages [61]. Prevention programs should guide oral health professionals to search for and identify precancerous lesions. The most common premalignant lesions are leukoplakia, erythroplakia, and oral lichen planus [68].

#### 3.5.1. Leucoplakia

Leucoplakia is characterized by white spots or plaques that are not scraped off. In the clinical diagnosis, leukoplakia must be differentiated from other lesions, such as lichen planus, candidiasis, friction keratosis, smoker’s keratosis, nicotinic and uremic stomatitis, leukoedema, and hairy leukoplakia [68]. Leucoplakia has been reported to have a malignant transformation rate between 1 and 5%; however, studies suggest that it may have a chance of dysplasia or invasive carcinoma [69,70].

#### 3.5.2. Erythroplasia

Erythroplasia is characterized by the presence of a red patch or spot. It is considered a high-risk lesion for malignancy and must be differentiated from erythematous candidiasis, erythema migrans, lichen planus lesions, lupus, and other erosive lesions [71,72]. In contrast to leukoplakia, erythroplakia is more than 90% likely to have dysplasia or carcinoma in situ [73].

#### 3.5.3. Lichen Planus

Lichen planus is an inflammatory disease from an unclear cause that presents as white reticular lesions that may be associated with atrophic, erosive, ulcerative, and plaque-like areas [74]. The pathophysiology of lichen planus begins with an autoimmune response mediated by T lymphocytes that causes alterations in the basal cells of the epithelium, generating an inflammatory infiltrate in the basement membrane and leading to subsequent complications [75].

### 3.6. Histological Aspects of Oral Cancer

Oral cancer presents various degrees of histological differentiation. Histologic features include (1) loss of basement membrane and stratum basal structure, (2) increased number of mitoses, and (3) invasion of the underlying connective tissue [75,76]. Oral cancer is the result of genetic and epigenetic changes that lead to histological changes, all associated with a malignant transformation of the epithelium [77,78]. It is an invasive neoplasm that has a poor prognosis and can develop metastases in distant organs. Oral cancer metastases spread primarily through the submandibular, cervical, and jugular lymph nodes. Distant metastasis spreads to the jaw and finally to the lungs, which compromises the health of the patient and has a higher risk of death [17,18,19,20]. Investigating and identifying the major mechanisms involved in oral carcinogenesis is therefore important.

### 3.7. Carcinogenesis of Oral Cancer

Carcinogenesis is a process that involves the alteration of molecular function, changes in cell morphology, and epithelial, connective tissue, and immune function. As a result of this process, cells acquire new functions that allow them to survive and proliferate [79]. Carcinogenesis comprises three phases: initiation, promotion, and progression. During initiation, endogenous and exogenous factors generate molecular defects, such as mutations, chromosomal abnormalities, and epigenetic alterations [80]. The initiation phase continues through the promotion phase, which is characterized by the selective proliferation of tumor cells with stem cell characteristics. Then, the progression phase continues, in which tumor cells act on their tumor microenvironment to create conditions that favor cell proliferation and survival [81]. In the carcinogenesis of oral cancer, oncogenes and tumor suppressor genes and their associated epigenetic alterations play an important role [82].

#### 3.7.1. Oncogenes and Tumor Suppressor Genes

Proto-oncogenes encode the proteins that regulate cell division and differentiation and activate oncogenes through DNA mutations and the inactivation of tumor suppressor genes [83]. Various oncogenes have been implicated in oral cancer development, including the epidermal growth factor receptor (EGFR/c-erb 1), members of the Ras gene family, c-myc, int-2, hst-1, PRAD-1, and bcl-1 [84]. Gain-function mutations of these oncogenes facilitate the appearance of neoplastic transformation. On the other hand, tumor suppressor genes participate in oral carcinogenesis and their alteration indicates the onset of a neoplastic transformation. Tumor suppressor genes associated with oral cancer include: (1) cell cycle genes (TP53, CDKN2A, and Rb1), (2) genes related to the tumor microenvironment, (3) adhesion molecules, and (4) DNA repair genes and genes associated with apoptosis [84].

#### 3.7.2. Epigenetic Alterations

DNA methylation mechanisms and histone code modifications may contribute to tumor development and neoplastic transformation [85]. Aberrant hypermethylation of the promoter regions of tumor suppressor genes disrupts the binding of a transcription factor, causing the genes to be silenced and promoting uncontrolled cell proliferation. The p16 protein, whose gene is located at the CDKN2A locus, is no longer expressed in 50–75% of oral cancer patients [86]. The absence of its expression is related to the methylation of the promoter of this gene, which affects the expressions of p15 and p14, two inhibitors of cyclin/CDK complexes. Gene silencing has also been detected in patients with oral cancer associated with DNA repair, apoptosis, the Wnt signaling pathway, and E-cadherin [86].

Histones are structural proteins that form a complex with DNA, and the acetylation or methylation of these proteins induces conformational changes in DNA that regulate transcription [87,88,89].

## 4. Treatment

Oral cancer treatment focuses on eradicating the tumor, preserving or restoring the shape and function of the mouth, preventing recurrence, and reducing the mortality rate while increasing the quality of life of the patient [21,22,23,90]. The main treatment option is the surgical resection of the tumor in combination with radiation therapy and chemotherapy, and it is most effective when oral cancer is detected early on. The success of treatments is significantly improved when performed by a multidisciplinary team, including maxillofacial surgeons, oral pathologists, oncologists, plastic surgeons, dentists, physiotherapists, radiologists, psychologists, and nutritionists [91].

### Prevention and Antioxidants

To diagnose premalignant lesions in the oral cavity in a timely manner, it is necessary to make an accurate diagnosis, as these lesions are usually not detected at an early stage. However, it is essential to identify the risk factors associated with the development of oral cancer. Preventing and controlling oral cancer is a global challenge; therefore, it is necessary to establish prevention programs to reduce the incidence of cases [92]. Oral cancer-prevention measures include self-examination, regular visits to the dentist, and eliminating risk factors. In these prevention programs, it is recommended to include a diet rich in foods with anti-inflammatory or antioxidant value. Optimal nutritional intake is a fundamental element for the preservation of health in general. It has been identified that some foods, such as fruits and vegetables, which are frequently found in the diet can have this type of protective effect [93,94]. Reports in recent years have investigated the beneficial effects of berries with special antioxidant properties in oral cancer [95]. In addition, in recent years, it has been suggested that a diet rich in fruit and vegetable phytochemicals can help reduce the risk of oral cancer [96,97].

## 5. Phytochemical Compounds

**Figure 3:**
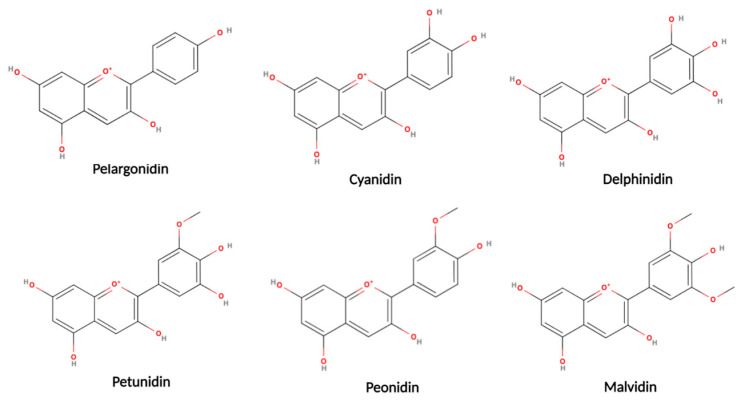
Representative chemical structures of blueberries and cranberries (created with the MolView program).

**Figure 4:**
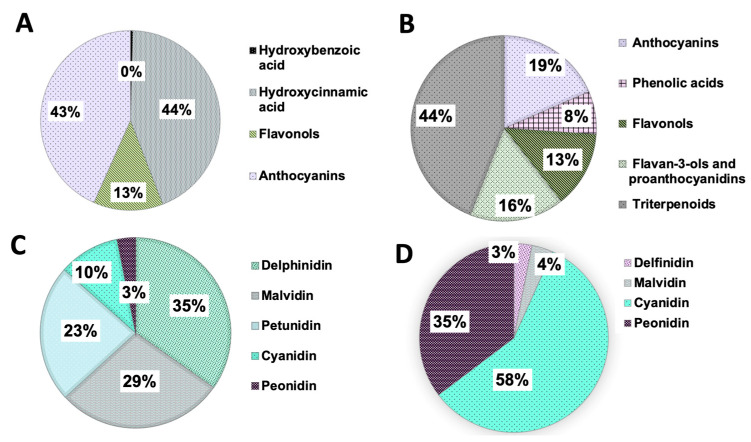
Percentage of some secondary metabolites in blueberries (fresh weight) and cranberries (dry matter) (**A**,**B**) and their anthocyanins (**C**) and derivatives (**D**). The percentages are calculated using the mean between the lowest and highest values identified in the literature for blueberries in mg/kg of fresh weight and in the case of the lingonberries in mg/100 g of dry matter, as referenced in the literature [98,99,132,133]. The values of each secondary metabolite depend on the size of the fruit, the state of maturation and, other postharvest conditions.

The phytochemicals or secondary metabolites of plants form a group of compounds that, although they are not considered essential nutrients, have beneficial properties for health when they are included in the diet. These compounds provide anti-inflammatory and antioxidant properties [15,16,17,98,99]. Approximately 10,000 plant-derived compounds are known in the world; however, only 200 plant species are considered safe for human consumption [98,99,100]. The Western diet, characterized by a high consumption of refined sugars, saturated fats, and red meat, combined with the inadequate consumption of fruits and vegetables and other risk factors, is associated with a higher incidence of oral and pharyngeal cancers [101]. Therefore, the consumption of phytochemical compounds through the diet can represent a strategy to reduce the negative effects of cancer risk factors associated with oral cancer and can be found in the *Ericaceae family*, especially in berries [94] (Figure 3 and Figure 4).

### 5.1. Family Ericaceae

The *Ericaceae* family is a family of plants composed of at least 4250 species distributed in 124 genera and nine subfamilies [102]. Within this family is the subfamily *Vaccinioidae*, which includes many important blueberry species, such as cranberries (*Vaccinium macrocarpon* A.), blueberries (*Vaccinium corymbosum* L.), European blueberries or bilberries (*Vaccinium myrtillus* L.), among others [103].These fruits contain a high concentration of phytochemical compounds; in particular, blueberries have twice the antioxidant capacity of red pomegranate [98]. In the last two decades, the therapeutic use of berry-derived compounds in the treatment of cancer has increased due to the association between the development of cancer and the consumption of fruits and vegetables [99,100,104]. In the studies conducted by Greenwald, new approaches have been discovered based on the interactions between the components of the diet, nutrients, genes, and environmental and dietary factors for the benefit of health [105]. It is important to note that the compounds responsible for biological activity in the *Ericaceae* family are anthocyanins, flavonoids, anthocyanidins, flavanols, flavan-3-ols, proanthocyanidins, phenolic acids, and triterpenoids [101,102,106,107,108].

### 5.2. Anthocyanidins

Anthocyanidins belong to the flavonoid family and anthocyanidins are the largest group of water-soluble pigments in the plant kingdom. The main sources of these phytochemicals are blueberries, cherries, raspberries, strawberries, etc., which usually give the characteristic color to this type of fruit [109]. These anthocyanins exhibit a wide range of biological properties, including antioxidant, anti-inflammatory, antimicrobial, and antitumor activities [110]. The ability of anthocyanins to (1) inhibit cell proliferation, (2) induce apoptosis by the intrinsic and extrinsic pathways, (3) suppress angiogenesis, (4) suppress angiogenesis through inhibition, (5) inhibit matrix metalloproteinase (MMP) expression, and (6) suppress plasminogen activator urokinase has been reported in in vitro and in vivo studies of hydrogen peroxide and tumor necrosis factor (TNF) induced by the expression of vascular epidermal growth factor (VEGF) in epidermal keratinocytes [111,112,113,114,115,116].

### 5.3. Anthocyanins

The purple, red, and blue colors in vegetables are due to anthocyanins. Fruits, such as purple cabbage, grapes, and blueberries, are an available food source with this property. Anthocyanins are the product of glycosidic replacement in anthocyanidins. Within the group of anthocyanins are delphinidin, malvidin, petunidin, cyanidin, and peonidin, together with their fractions of glucose, galactose, and arabinose. These anthocyanins are found in berries. They have been reported to present antitumor activity against oral cancer [99,117,118,119,120,121,122,123,124,125]. The anthocyanins of black rice (*Oryza sativa*) have anticancer properties, such as the inhibition of metastasis of the oral cancer cell line (CAL-27), through the downregulation of matrix metalloproteinases [126]. In addition, a similar effect was observed in other oral cancer cell lines using a lyophilized extract of *Rubus idaeus*, which showed a concentration-dependent inhibition of both the migration and invasion of the oral cancer cell line SCC-9 and SAS [127]. Blueberry anthocyanins may induce G2/M cell cycle arrest in oral cancer cell line KB [128]. In addition, anthocyanins also increase the levels of caspase 9 and cytochrome c in KB cells, which indicates the induction of apoptosis, and simultaneously increase the amount of p53, which in most neoplasms has lost its function due to mutation [128]. Moreover, certain strawberry-derived anthocyanins, including cyanidin-3-glucoside (C3G), pelargonidin, and pelargonidin-3-glucoside (P3G), have been shown to inhibit tumor growth in oral cancer cell lines in the colon and prostate [129].

### 5.4. Flavan-3-ols and Proanthocyanidins

Flavan-3-ols represent one of the most complex subclasses of flavonoids, which range from monomers to oligomeric and polymeric proanthocyanidins, also named condensed tannins [130]. In studies on esophageal adenocarcinoma, the antiproliferative effect of protoanthocyanidins was observed, showing their ability to stop the cell cycle, induce apoptosis, or trigger autophagy as an alternative mechanism involving PI3K (phosphoinositide-3-kinase), AKT (Protein kinase B) and the mammalian target of rapamycin (mTOR) signaling [131]. Catechin, epicatechin, and polymeric pro-anthocyanidins are found in cranberries. It is known that this type of compound can delay the onset of tumors in transgenic mice that spontaneously develop tumors [20].

### 5.5. Phenolic Acids

Phenolic acids belong to a broad family of phenolic molecules, which are subdivided into hydroxybenzoic and hydroxycinnamic acids. Each of these compounds has shown antioxidant, antiproliferative, and anti-inflammatory activities [20]. Cranberries and blueberries contain phenolic compounds with antitumor potential [104].

### 5.6. Triterpenoids

Triterpenes are natural alkenes that are composed of 30 carbon atoms and are made of six isoprene units. These are usually found in a linear fashion, mainly in the form of squalene derivatives, tetracyclic and pentacyclic, containing four and five cycles, respectively, as well as those with two or three cycles [106,107]. In cranberries, ursolic, oleanolic, and betulinic acids are found in greater proportions [99]. These acids have antitumor, anti-inflammatory and antioxidant activities [122]. The compounds responsible for the biological activity in the *Ericaceae* family are anthocyanins, flavonoids, anthocyanidins, flavanols, flavan-3-ols, proanthocyanidins, phenolic acids, and triterpenoids, as shown in Table 1.

## 6. Berries and Cancer

Berries are rich in minerals, vitamins, fatty acids, fiber, and polyphenolic compounds, including pterostilbene, malvidin, and malvidin-3-galactosidase [20,130]. Cranberries and blueberries have been shown to have antitumor properties in vitro, in vivo, and in clinical studies [117,118,119]. This activity is due to the presence of compounds, such as phenolic acids, flavonoids, anthocyanins, procyanidins, ascorbic acid, quercetin, kaempferol, catechin, epicatechin, p-coumaric acid, gallic acid, caffeic acid, ferulic acid, hydroxycinnamic acid, and chlorogenic acid, which are able to inhibit proinflammatory molecules, decrease oxidative stress, prevent DNA damage, inhibit tumor cell proliferation, and enhance tumor cell apoptosis Table 2 [120,121]. Chlorogenic acid, which is found in a variety of fruits, including blueberries, has been studied for its potential health benefits, particularly for its antioxidant and potential antitumor properties. The effects of coffee consumption on antitumor activity are complex and multifaceted. This is because coffee contains a variety of compounds that have been studied for their potential health effects [134]. Despite its high antioxidant content, the processing and heating of coffee may reduce its antitumor activity [135]. Further investigations are needed to understand the specific mechanisms by which coffee exerts its potential antitumor effects. Seeram determined that the polyphenolic compounds present in cranberry were responsible for the antiproliferative effects found in oral, prostate, and colon cancer cells, not the sugars [118]. These findings have been extended to other widely consumed berries. These include blackberries, black raspberries, blueberries, red raspberries, and strawberries [119]. Protoanthocyanidins derived from V. macrocarpon were reported to be the relevant bioactives involved in the reduction in urinary tract infections after cranberry juice consumption due to the inhibition of the adhesion of *E. coli fimbriae* to uroepithelial cells [136]. Furthermore, when these protoanthocyanidins were used in tumor explants of colon or prostate cancer cells, a decrease in the growth rate was observed in vivo [137]. Therefore, it is important to apply this knowledge to models of oral cancer and to strengthen epidemiological studies that assess the benefit of berries to reduce the incidence of oral cancer.

### 6.1. Mechanism of Action of Berries in Cancer

**Figure 5:**
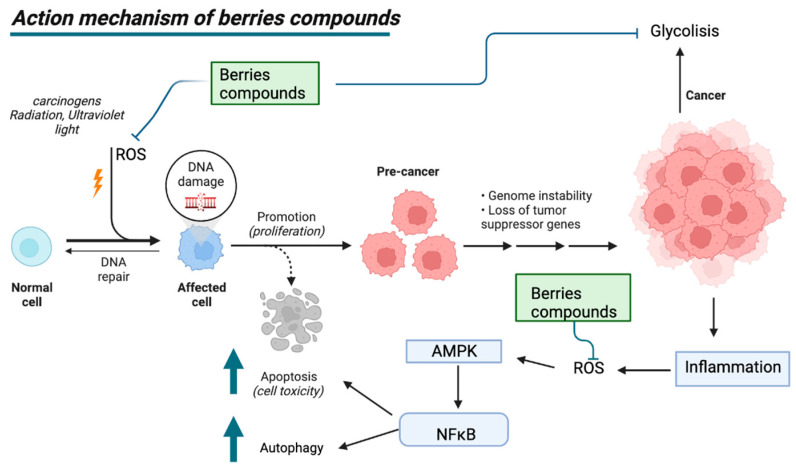
Mechanism of action of berries in cancer. Created with BioRender.com (accessed on 10 March 2022).

The mechanism of action of berry-derived phytochemicals can induce various effects on tumor cells, such as the inhibition of the nuclear transcription factor (NF-κB), inhibition of the MAP kinase pathway, interference with the production of detoxification enzymes, interference with the beta-catenin modulation of apoptosis, and modulation of the PI3K/AKT/mTOR pathway [138,139]. Studies have reported that polyphenols have an action on the NF-κB pathway, with effects on the Mitogen-activated protein kinase (MAP kinase pathway), the cAMP-dependent protein kinase (PKA) pathway, apoptosis, and the generation of oxidative stress, processes that depend on the consumption of berries in the diet [139,140]. The physicochemical and biotransformation properties of berry polyphenols by liver enzymes and the intestinal microbiota exert different effects on tumor cells in in vitro and animal models [141,142]. Resveratrol is the most widely characterized example of polyphenols regarding its cellular effects. This compound, found in red grapes, can have effects on tumor cells, in addition to inducing cell cycle arrest in oral cancer lines, and it can induce DNA damage and increase cell death in several cancer models [143,144]. Resveratrol appears to influence autophagy, as an alternative mechanism, and apoptosis, affecting oral cancer cell resistance to cisplatin through the AMPK and PI3K/AKT/mTOR pathways [145,146]. In esophageal adenocarcinoma, the anti-proliferative effects of resveratrol proanthocyanidins were observed, in addition to their ability to arrest the cell cycle [147]. Blackberries have also been reported to inhibit oxidative stress in rat esophageal squamous cell carcinogenesis models and the hydrogen peroxide-activated NF-kB/MAPK pathway [148]. In a study, a potential mechanism of oral cancer inhibition was identified through black raspberries via the glycolysis and AMPK pathways [149]. The authors suggested administering blackberries to animals to prevent oral cancer. Black raspberry phytochemicals can negatively modulate these pathways and thus compromise tumor cell growth (Figure 5) [149]. Berry flavonoids and polyphenols have been associated with effects on inflammation, apoptosis, autophagy, and the inhibition of the PI3K/Akt/mTOR pathway, among others, in cancer models [149]. The effects on the immune system of berries have shown effects on cancer metabolism in mice against the cell cycle, activation of MAP kinase signaling, DNA repair, leukocyte extravasation, and modulation of the inflammatory response [150].

### 6.2. Mechanism of Action of Berry-Derived Phytochemicals in Oral Cancer

The fruits of the species *Vaccinium* ssp. represent an excellent natural food resource rich in phenolic compounds, flavonoids, polyphenols, anthocyanins, procyanidins, chlorogenic acid, ascorbic acid, quercetin, kaempferol, catechin, epicatechin, p-coumaric acid, gallic acid, caffeic acid, ferulic acid, and hydroxycinnamic acid, among others [110]. The consumption of both fresh and processed products can be an alternative to prevent oral squamous cell cancer [151].

### 6.3. Consumption of Cranberries and Blueberries in Protection against Risk Factors for Oral Cancer

Preventive measures to protect against oral cancer are classified into three categories: primary, secondary, and tertiary [152]. Among these measures is chemoprevention, which consists of the administration of an agent to prevent the appearance of cancer [153]. These agents can be drugs or natural products that are easy to administer, with little or low toxicity, and that do not cause long-term sequelae. Blueberries are interesting candidates because they contain many phytochemicals with antitumor potential [154]. These phytochemicals can be effective in the short and long terms, and they can be consumed fresh or processed. The main source of phytochemicals in cranberries and blueberries is anthocyanins, which account for more than 60% of the total polyphenols in their ripe state. This means approximately 387–487 mg/100 g anthocyanins in their fresh form [155]. To take advantage of these effects, it is recommended to drink a 250 mL cup of fresh cranberry or processed juices daily for at least 28–36 days, both in the morning and afternoon [156,157,158,159]. However, it is important to note that the processed juice should not contain ascorbic acid, as it reduces the number of phenolic compounds present in the juice and its biological efficacy by up to 10% [160]. Cranberries and blueberries are rich in phytochemicals with anticancer potential, such as anthocyanins and flavanols [161]. These phytochemicals can be consumed both fresh and processed to exert their potential long-term protective effects against oral cancer. Other important components in blueberries, especially cranberries, are flavan-3-ol and triterpenoids. These phytochemicals can also contribute to the treatment of oral premalignant lesions and the reduction in other factors that promote oral carcinogenesis. It is important to mention that the effects of climate change on secondary metabolism cover many aspects, ranging from the molecular level to the overall effects on organisms resulting from changing concentrations of phytochemical compounds. To obtain the essential information needed to understand how plants perform under changing climatic conditions, a thorough analysis of the existing knowledge on the effects of climate change components on berry secondary metabolites is crucial. Further research is needed to better understand the complex effects of multiple environmental factors on berry secondary metabolites [162]. However, there are still a relatively small number of studies investigating the metabolome in berries. Berries, as a group of fruits, have remarkable resilience in the preservation of their inherent qualities. Cranberries are widely recognized for their ability to retain their distinctive biochemical characteristics [163].

#### 6.3.1. Cranberries and Blueberries and Their Protection against Tobacco-Induced Oral Cancer

A variety of oral lesions, including leukoplakia, erythroplakia, oral submucosal fibrosis, and oral cancer, have been implicated in tobacco use [164]. This is due to the presence of over sixty carcinogens in the smoke of cigarettes, as well as, at minimum, sixteen in uncombusted tobacco [91]. This includes tobacco-specific nitrosamines, such as 4-(methylnitrosamine)-1-(3-pyridyl)-1-butanone (NNK) and N’-nitrosonornicotine (NNN); polycyclic aromatic hydrocarbons, such as benzo[α] pyrene; and aromatic amines, such as 4-aminobiphenyl, which have been shown to cause cancer [165]. Strategies to use chemopreventive agents to counteract or inhibit the effects of tobacco use on oral carcinogenesis can be very useful in preventing this disease. One example of these agents is blueberry extracts, which have been shown in an in vivo study to inhibit the initiation and progression of carcinomas through the inhibition of the TGF-β and PI3K/AKT pathways [166,167]. Furthermore, supplementation suppressed the activation of NF-κB, preventing the translocation of this transcription factor [168]. Furthermore, these extracts have been shown to modulate the expression of oncomiR miR-21 and the tumor suppressor let-7 [167]. A study on a mouse model showed that black raspberry extracts inhibit the binding of the carcinogen dibenzo [α, l] pyrene-DNA, which is crucial for the repair of lesions caused by this carcinogen in DNA [169]. Protocatechuic acid, found in blueberries, has also been shown to be responsible for some of the benefits of anthocyanin consumption, including the inhibition of mutagenic effects as well as the generation of DNA adducts of the tobacco smoke carcinogen dibenzopyrene [94].

#### 6.3.2. Cranberries and Blueberries and Their Protection against Alcohol-Induced Oral Cancer

Alcohol consumption, such as tobacco smoking, has been recognized as an important contributor to the development of oral cancer for over fifty years. It has been associated with approximately 75% of cancers affecting the superior aerodigestive tract and causes changes in the cellular structure of the oral epithelium [170]. Mechanisms through which the consumption of alcohol causes carcinogenic damage are not fully understood; however, they may include the genotoxic effect of acetaldehyde on the oral mucosa, an increase in the concentration of estrogens, the role of a solvent producing other carcinogens, such as those from tobacco, the generation of ROS and nitric oxide synthases (NOS), and alterations in folate metabolism [171]. It is believed that the consumption of blueberries may have chemopreventive effects on oral cancer induced by alcohol consumption due to its content of phytochemicals, such as quercetin and resveratrol [172]. Anthocyanins, which are one of the major components of blueberries, have interesting anticancer properties that are useful for the prevention of oral cancer [116]. One of these compounds, malvidin (malvidin-3-glucoside), showed inhibitory activity against (Signal transducer and activator of transcription 3) STAT-3 within the oral cancer cell line (SCC131) by suppressing the phosphorylation and nuclear translocation of this factor, which resulted in cell cycle arrest and mitochondrial-mediated apoptosis [120]. In addition, blueberry extract has been shown to inhibit The Janus-Kinase signal transducer and the transcription activation pathway (JAK/STAT-3) signaling by modulating the downstream sites affecting cell proliferation and apoptosis in a hamster model of oral oncogenesis [120]. Therefore, due to the action of this phytochemical, it is possible to inhibit oral carcinogenesis by alcohol, which is an important inducing factor of this type of cancer that can contribute to the large number of people who regularly consume this substance [173,174]. Other studies have shown the chemopreventive capacity of grape wine in cells of the oral mucosa. Although regular consumption in moderate doses produces metabolites, such as acetaldehyde, which is the main producer of DNA adducts, it has been shown that grape wine has the ability to mitigate the deleterious actions of alcohol and reduce the chances of developing oral cancer [124,175]. This is due to the presence of phenolic compounds, such as those found in cranberries, which activate the p53 tumor suppressor gene to induce cell cycle arrest as well as apoptosis in cells of the oral cavity [119].

### 6.4. Consumption of Cranberries and Blueberries Protects against the Effects of Bacteria and Poor Oral Hygiene

The oral cavity is a special place where there are more than 250 varieties of microorganisms called commensals, which have a crucial role in maintaining the individual status of organisms [176]. Many of these species, according to epidemiological studies, are closely related to oral cancer. Among the most important are *Fusobacterium nucleatum* and *Porphyromonas gingivalis*, among others, which have been related to different types of carcinomas and oral tumor processes [177]. Additionally, some papillomaviruses, oral fungi, such as *Candida albicans*, and parasites have been linked to oropharyngeal cancer [178]. *Candida albicans* infection has been implicated in the initiation and progression of oral cancer through the activation of proto-oncogenes, induction of DNA damage, and overexpression of oncogenic pathways [179]. Inflammatory signaling in the oral mucosa can also be modulated by the phytochemicals of blueberries, especially red berries. The crude extracts and their fractions enriched with other species of red fruits (*Vaccinium myrtillus* L. and *Malpighia punicifolica* L.) exert antiadhesion activity against *Candida* spp. when used at concentrations > 1.25 mg/mL [180]. This mechanism is related to the action of type-A cranberry proanthocyanidins, which do not have a relevant effect on *Candida* spp. but do so on the adhesion of this on the oral mucosa [181,182]. Thus, for carcinogenic implantation functions to occur, phenomena, such as microbial dysbiosis, colonization, and translocation, must occur, which produce an excessive inflammatory response, host immunosuppression, enhancement of malignant transformation, anti-apoptotic effects, and the secretion of carcinogens [178]. Therefore, the consumption of blueberries and cranberries may be a potential source to prevent these types of infections [183]. An example of this is the effect of proanthocyanidins (PACs) in both blueberries and cranberries to reduce the deleterious effects of the *Porphyromonas gingivalis* species on the cells of the oral mucosa [184]. This is achieved by protecting against the damage produced by said bacteria in the keratinocytic gingival barrier, inhibiting its translocation, reducing the proteolytic degradation of tight epithelial junctions, and reducing the secretion of IL-6 and IL-8, among others, in an in vitro model of the gingival keratinocyte barrier against *P. gingivalis* [185]. In addition, PACs from cranberries have also been associated with antimicrobial, anti-adhesion, antioxidant, and anti-inflammatory properties, which is why they can be potentially useful in the prevention of periodontal disease that affects dental tissues [186]. This is achieved through the inhibition of both bacterial and host-derived proteolytic enzymes, as well as host inflammation and osteoclast differentiation and activity [186].

### 6.5. Cranberries and Blueberries and Their Protection against Oral Cancer Induced by Viral Infections

Two infectious factors associated with the onset and development of oral cancer are human papillomavirus (HPV) and Epstein–Barr virus (EBV) [187,188]. For example, the polyphenolic and flavonoid components (also found in cranberries and blueberries) in the *Polygonatum odoratum* plant have been shown in recent studies to have antibacterial, antifungal, antioxidant, anti-inflammatory, and anticancer activities. A study has shown that extracts of this plant significantly affect human lymphoblastoid carriers of the Epstein–Barr virus genome through the induction of apoptosis, cell cycle arrest, inhibition of cell proliferation, migration, and colonization [189]. Moreover, this extract suppresses proteins that are essential to cell proliferation, colonization, and migration, including cyclin D, cyclooxygenase 2 (COX-2), matrix metalloproteinase-9 (MMP-9), and vascular endothelial growth factor A (VEGF-A) [188]. This makes these components of interest in the prevention of oral cancer mediated by this virus.

**Table 2:** Effects of cranberry extracts and phytochemicals against oral cancer.

Cranberry Type	Type of Study Conducted (In Vitro/In Vivo/Clinical Study)	Evidence against Oral Cancer	Reference
***Vaccinium corymbosum* L.** (blueberries)	In vitro	The methanolic extract of blueberries inhibits cell proliferation in the oral cancer line KB.	[128,188]
	In vivo/In vitro	Dietary administration of blueberry produces significant effects on the SCC131 cancer cell line through the inhibition of TGF-β and NF-κB, as well as act against invasion and angiogenesis at doses higher than 200 mg/kg.	[20,167]
	In vitro	The phytochemical pterostilbene present in blueberries induces apoptotic cell death and, through autophagy in cisplatin-resistant human oral cancer cells (CAR cells), which is related to the AKT pathway, are mediated by the suppression of MDR1.	[100]
***Vaccinium macrocarpon* A.** (lingonberries)	In vitro	The methanol extract of the cranberries inhibits cellular proliferation in the line of oral cancer KB.	[128,188]
	In vivo/In vitro	The extract composed of proanthocyanidins (C-PAC) derived from cranberries inhibits the growth of resistant and acid-sensitive esophageal adenocarcinoma (EAC) cells, both in cell lines and xenotransplant mice, inducing caspase-independent cell death, mainly by the autophagic pathway.	[131]
	In vitro	The hydroethanolic extract of cranberries produces an antiproliferative effect on the caspase-independent KB cell line, mainly by the autophagic route.	[190]
	In vitro	Cranberry extract produces an inhibitory effect on the proliferation of OSCC lines cAL27 and SCC25 at an optimal concentration of 40 μg/mL, producing the upstream regulation of caspases 2 and 8, and effects cell adhesion, cell morphology, and the cell cycle.	[191]

## 7. Limitations and Perspectives of the Consumption of Cranberries and Blueberries for Protection against Oral Cancer

Diet is a complex issue influenced by cultural and resource-related factors. Scientific research aimed at understanding the molecular mechanisms underlying the reduced risk of cancer associated with certain dietary habits has revealed that isolating individual compounds fails to capture the broad range of benefits observed in epidemiological studies. Allium vegetables, including garlic, onions, chives, shallots, and leeks, present an intriguing case. This diverse group of plants comprises over 500 species and contains numerous bioactive compounds, particularly sulfur compounds, such as allicin, S-allylcysteine, diallysulfides, and others. Assessing the impact of allium consumption remains challenging due to the likely influence of various environmental and dietary variables on its potential cancer-preventive effects. However, several phytochemicals found in allium vegetables have demonstrated their ability to reduce cancer risk at different stages of cancer development, including initiation, promotion, and progression. Similar findings have also been observed in the context of berries and fruits. The scientific evidence supports the notion that lifestyle modifications, particularly dietary changes, can enhance cancer-prevention efforts. However, it is important to avoid overstating the significance of individual components, and further studies are warranted to expand our understanding [192].

### Limitations

Oral cancer is a multifaceted disease involving various factors that trigger the carcinogenic process. Strategies incorporating antioxidants and phytochemicals from cranberries can help prevent these factors by modifying molecular mechanisms and inducing apoptosis in tumor cells. However, it is crucial to consider the source of blueberries (natural or processed) as the latter may degrade phytochemicals, reducing their protective effects. Furthermore, there is a lack of clinical research investigating the impact of cranberries and blueberries on oral cancer through traditional consumption methods (juice and fresh) among smokers and regular alcohol consumers to determine their protective effects. The effectiveness of cranberry supplementation also depends on the stage of carcinogenesis, with varying benefits depending on tumor development levels. Despite the existing evidence, scientific studies supporting the use of these fruits for oral cancer prevention and treatment are limited. Additional research is needed to recommend their consumption, particularly in advanced stages of oral tumor diseases, such as metastasis, where the effects remain unknown. Blueberries contain phytochemicals, such as anthocyanins, quercetin, and ellagic acid, which similarly reduce the diet-related risk factors associated with oral cancer, akin to black raspberries. It is important to be aware of these limitations in the interpretation of the available published scientific evidence on the association between berries and oral cancer reduction.

Perspectives: conducting rigorous clinical trials with carefully designed protocols and representative samples is a promising approach to berry research for reducing oral cancer risk factors. To develop precise treatment strategies, it is critical to understand the specific mechanisms by which the bioactive compounds in berries exert their beneficial effects. In addition, there is great promise for increasing efficacy and improving outcomes by exploring combinations of berries with other treatments. Elucidating the mechanisms of action and exploring berry combinations with other treatments to improve outcomes are future perspectives in this field.

## 8. Conclusions

Studies performed in vitro, in vivo, and in clinical research have shown that the phytochemicals and polyphenolic compounds found in blueberries, cranberries, and other berries have antitumor properties. Polyphenolic compounds found in cranberries are responsible for the antiproliferative effects on oral cancer cells in in vitro and in vivo models. Berries’ mechanisms of action in oral cancer include: (i) the inhibition of inflammation through interference with the nuclear transcription factor NF-κB, (ii) effects on proliferation by interfering with the MAP kinase pathway, (iii) reduction in resistance by interfering with the expression of detoxification enzymes, (iv) interference with the β-catenin signaling pathway, (v) modulation of apoptosis, and (vi) modulation of the antiapoptotic and anabolic pathways of PI3K/AKT/mTOR. The use of protective strategies is based on the consumption of functional foods, nutraceuticals, or supplements based on fruits, such as berries of the species *Vaccinium* ssp. It can be of great interest to reduce the effects of the risk factors associated with oral cancer.

## Figures and Tables

**Table 1:** Secondary metabolites present in cranberry and blueberry extracts. * Note 1: dm—dry matter; fw—fresh weight. Note 2: The concentration of each secondary metabolite depends on the size of the fruit, the state of maturation, the postharvest conditions, and the environmental and storage conditions.

Secondary Metabolite	Average Concentration in the Extract *	Reference:
***Vaccinium corymbosum* L. (blueberries)**		
***Phenolic acids***		
**Hydroxybenzoic acid**	1.5 mg/kg fw	[132]
**Hydroxycinnamic acid**	135 mg/kg fw	[132]
***Flavonoids***		[132]
**Flavonols**	38.7 mg/kg fw	[132]
**Anthocyanins**	134 mg/kg fw	[132]
***Vaccinium macrocarpon* A. (lingonberries)**		
**Anthocyanins**	695–1716 mg/100 g dm	[99]
**Phenolic acids**	327–649 mg/100 g dm	[99]
**Flavonols**	643–1088 mg/100 g dm	[99]
**Flavan-3-ols and proanthocyanidins**	860–1283 mg/100 g dm	[99]
**Triterpenoids**	2528–3201.5 mg/kg dm	[99]

## Data Availability

Not applicable.

## Abbreviations

OSCC	Oral squamous carcinoma cell
HPV	Human papillomavirus
EBV	Epstein–Barr virus
NNN	N′-nitrosonornicotine
NNK	4-(methylnitrosamine)-1-(3-pyridyl)-1-butanone
ROS	Reactive oxygen species
BMI	Body mass index
EGFR/c-erb	Epidermal growth factor receptor
TNF	tumor necrosis factor
MMP	matrix metalloproteinase
CAL-27	oral cancer cell line
C3G	cyanidin-3-glucoside
PACs	Proanthocyanidins
COX-2	Cyclooxygenase 2
MMP-9	Matrix metalloproteinase-9
VEGF-A	Vascular endothelial growth factor A
NF-κB	Nuclear transcription factor
NOSs	Nitric oxide synthases
P3G	pelargonidin-3-glucoside
SCC131	oral cancer cell line 131
PI3K	phosphoinositide-3-kinase
AKT	Protein kinase B
mTOR	the mammalian target of rapamycin
MAP	Mitogen-activated protein kinase
PKA	cAMP-dependent protein kinaseB
AMPK	the AMP-activated protein kinase
TGF-β	Transforming growth factor-β
STAT-3	Signal transducer and activator of transcription 3
JAK/STAT	The Janus-Kinase signal transducer and the transcription activation pathway
